# Prevalence of Integrase Strand Transfer Inhibitors (INSTI) Resistance Mutations in Taiwan

**DOI:** 10.1038/srep35779

**Published:** 2016-10-25

**Authors:** Sui-Yuan Chang, Pi-Han Lin, Chien-Lin Cheng, Mao-Yuan Chen, Hsin-Yun Sun, Szu-Min Hsieh, Wang-Huei Sheng, Yi-Ching Su, Li-Hsin Su, Shu-Fang Chang, Wen-Chun Liu, Chien-Ching Hung, Shan-Chwen Chang

**Affiliations:** 1 Department of Clinical Laboratory Sciences and Medical Biotechnology, National Taiwan University College of Medicine, Taipei, Taiwan; 2 Department of Laboratory Medicine, National Taiwan University Hospital and National Taiwan University College of Medicine, Taipei, Taiwan; 3 Department of Internal Medicine, National Taiwan University Hospital and National Taiwan University College of Medicine, Taipei, Taiwan; 4 Department of Medical Research, China Medical University Hospital, Taichung, Taiwan; 5 China Medical University, Taichung, Taiwan

## Abstract

Antiretroviral therapy containing an integrase strand transfer inhibitor (INSTI) plus two NRTIs has become the recommended treatment for antiretroviral-naive HIV-1-infected patients in the updated guidelines. We aimed to determine the prevalence of INSTI-related mutations in Taiwan. Genotypic resistance assays were performed on plasma from ARV-naïve patients (N = 948), ARV-experienced but INSTI-naive patients (N = 359), and raltegravir-experienced patients (N = 63) from 2006 to 2015. Major INSTI mutations were defined according to the IAS-USA list and other substitutions with a Stanford HIVdb score ≧ 10 to at least one INSTI were defined as minor mutations. Of 1307 HIV-1 samples from patients never exposed to INSTIs, the overall prevalence of major resistance mutations to INSTIs was 0.9% (n = 12), with an increase to 1.2% in 2013. Of these 12 sequences, 11 harboured Q148H/K/R, one Y143R, and none N155H. Of 30 sequences (47.6%) with INSTI-resistant mutations from raltegravir-experienced patients, 17 harboured Q148H/K/R, 8 N155H, and 6 Y143C/R. Other than these major mutations, the prevalence of minor mutations were 5.3% and 38.1%, respectively, in ARV-naive and raltegravir-experienced patients. The overall prevalence of INSTI mutations remains low in Taiwan. Surveillance of INSTI resistance is warranted due to circulation of polymorphisms contributing to INSTI resistance and expected increasing use of INSTIs.

Integrase strand transfer inhibitor (INSTI)-based regimens are recommended as first-line combination antiretroviral therapy (cART) in several updated treatment guidelines for HIV after the efficacy of the three INSTIs (raltegravir, elvitegravir, and dolutegravir) has been demonstrated in randomized clinical trials^1^,^2^,^3^,^4^,^5^,^6^,^7^,^8^. INSTI-containing regimens have good tolerability and safety profiles and less concerns about CYP 3A4-associated drug-drug interactions, with exception of elvitegravir when combined with cobicistat, for most patients.

With the widespread use of INSTIs, surveillance of the prevalence of INSTI resistance among ART-naive patients is critical in optimizing ART efficacy, given the concerns that INSTI, particularly raltegravir and elvitegravir, have a relatively lower genetic barrier to emergence of resistance mutations when compared with boosted PIs^9^. With one or two major mutations, such as Q148HKR ± G140SA, N155H ± E92Q or Y143CR ± T97A, a marked reduction of viral susceptibility to raltegravir and elvitegravir has been observed. According to recent publications from resource-rich countries such as the United States and Europe, transmitted INSTI resistance remains rare^10^,^11^,^12^. Nevertheless, the prevalence of transmitted INSTI resistance may increase in the next several years when the use of INSTI-containing ART increases. In addition, polymorphisms in integrase, which might modulate the efficacy of raltegravir and elvitegravir, have been reported and observed in patients receiving INSTI-containing regimens with virological failure. An increased frequency in HIV-1 integrase polymorphisms in patients with treatment failure and/or patients with reverse-transcriptase resistance mutations was reported^13^. In this study, we aimed to determine the prevalence of INSTI-resistant mutations in ART-naive patients, and to compare the prevalence of INSTI-resistant mutations/polymorphisms between ART-naive, ART-experienced/INSTI-naive, and raltegravir-experienced patients in Taiwan, where raltegravir was first introduced into clinical use in 2009.

## Results

### Characteristics of study population

From June 2006 to October 2015, a total of 1370 non-duplicated blood specimens were subjected to genotypic analysis of INSTI resistance at the National Taiwan University Hospital (NTUH). These specimens were from ART-naïve patients (N = 948), ART-experienced patients who had never been exposed to INSTIs (N = 359), and patients receiving raltegravir-containing regimens and experiencing virological failure (N = 63). Baseline characteristics of the patients included in this study are shown in Table 1. The INSTI-naive patients were predominantly males (95.8%); and 84.1% of them were men who have sex with men (MSM), 8.5% injecting drug users (IDU), and 6.8% heterosexuals. The majority of the patients were infected with subtype B viruses (85.7%), followed by CRF07_BC (9.0%) and CRF01_AE (4.9%). The mean baseline plasma HIV RNA load (PVL) was 4.74 log_10_ copies/mL (standard deviation [SD], 0.73 log_10_ copies/mL), with 33.1% of the patients having PVL > 5 log_10_ copies/mL. The mean CD4 count was 299 cells/mm^3^ (SD, 198 cells/mm^3^), with 31.7% having CD4 counts < 200 cells/mm^3^.

Compared to ART-experienced/INSTI-naive patients, ART-naïve patients had a higher percentage of male gender (97.1% vs 92.7%, *P* < 0.001), were younger (mean age, 31.4 years vs 37.2 years, *P* < 0.001) and were more likely to be MSM (87.2% vs 75.5%, *P* < 0.001) and infected with subtype B virus (87.0% vs 82.2%, *P* = 0.03), and to have higher mean CD4 count (315 cells/mm^3^ vs 256 cells/mm^3^, *P* < 0.001).

### INSTI-related mutations

**Figure 1:**
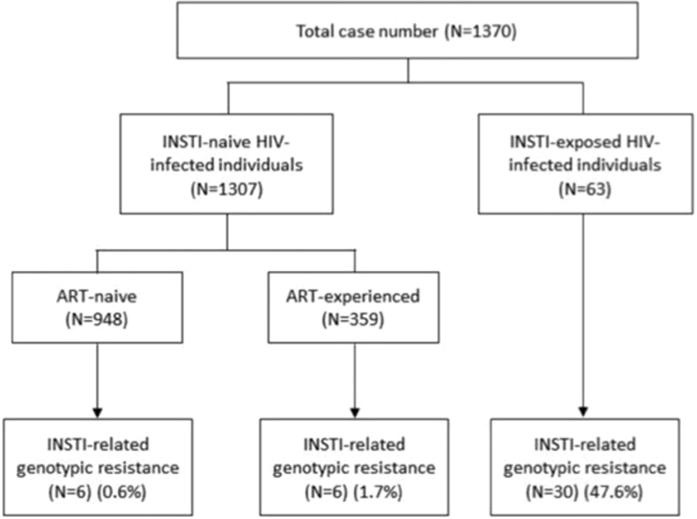
The flowchart of the study. (INSTI, integrase strand transfer inhibitor; ART, antiretroviral therapy).

Among the 1,307 INSTI-naive study subjects, 12 (0.9%) were infected with HIV-1 that exhibited major mutations shown to cause a marked reduction of viral susceptibility to raltegravir and elvitegravir (Fig. 1). The prevalence of INSTI-resistance mutations in ART-experienced/INSTI-naive patients was higher than that in ART-naive patients (1.7% v.s. 0.6%, *P* = 0.08). While the first case of INSTI-related resistance mutations was identified in a patient included in November 2006, the majority of the cases (n = 9) were identified in patients included in 2013. The prevalence of INSTI-related resistance mutations was <1% before 2012 [0.7% (1/136) between 2006 and 2011, 0.5% (1/200) in 2012], which increased to 1.2% (9/766) in 2013, and gradually declined to 0.5% (1/206) in 2014–15.

The 12 INSTI-resistant HIV-1 sequences were all of subtype B; 6 of them were from ART-naive and 6 from ART-experienced/INSTI-naive patients. The amino acid mutations/polymorphisms detected in these individuals are listed in Supplementary Table 1. Among these sequences, Q148H/K/R mutations were found in 91.7% (N = 11) and Y143R mutation in one; yet none of them harboured an N155H mutation. One sequence was identified to harbour Q148R/L74V/P145R mutation, which was predicted to have medium-level resistance to dolutegravir. The rest of the sequences only harboured a single major mutation without other relevant mutations and were predicted to have low-level resistance to dolutegravir.

**Figure 2:**
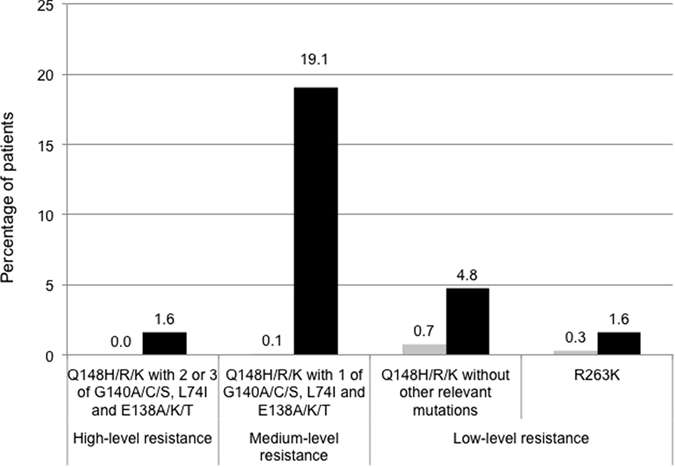
Predicted cross-resistance to douletagravir in INSTI-naive patients and INSTI-experienced patients. INSTI-naive patients and INSTI-experienced patients were labeled in grey bars and black bars, respectively. High-level of resistance to dolutegravir was defined as having Q148H/R/K with 2 or 3 of G140A/C/S, L74I and E138A/K/T; medium-level of resistance to dolutegravir was defined as having Q148H/R/K with 1 of G140A/C/S, L74I and E138A/K/T; low-level of resistance to dolutegravir was defined as having Q148H/R/K without other relevant mutations or having R263K mutation; no significant resistance to dolutegravir was defined as having none Q148H/R/K mutations.

The presence of INSTI resistance mutations was also determined in 63 INSTI-experienced patients. Thirty patients (47.6%) harboured INSTI-related major mutations, which included 17 with Q148H/K/R mutations, eight with N155H mutations, and six with Y143C/H/R mutations. The concurrent major/minor INSTI-related mutations in these sequences are summarized in Supplementary Table 2. Among the INSTI-experienced patients, only one (1.6%) had Q148H/R/K along with two or three of G140A/C/S, L74I and E138A/K/T mutations and were predicted to have high-level resistance to dolutegravir. Twelve sequences (19.1%) had Q148H/R/K with one G140A/C/S, L74I, and E138A/K/T, which were predicted to have medium-level resistance to dolutegravir (Fig. 2).

Beside major INSTI mutations, other integrase substitutions with a Stanford HIVdb score ≥ 10 to at least 1 INSTI, and some polymorphisms that have been reported in previous studies or observed in this study were also determined. Their overall prevalence in INSTI-naive patients and INSTI-experienced patients was 5.2% (69/1307) and 38.1% (24/63), respectively. In INSTI-naive patients, the resistance mutations included L74M (n = 28), E92V (1), Q95K (4), T97A (4), E138AK (3), Y143S (1), V151AL (1), N155S (9), E157Q (11), G163K/R (5), S230R (1) and R263K (4) (Supplementary Table 3). The prevalence of T97A, G140CAS, E157Q, and V151I was all significantly higher in specimens from raltegravir-experienced patients than those from INSTI-naïve patients. On the contrary, the prevalence of P145R was significantly higher in INSTI-naïve patients than in raltegravir-experienced patients.

### Transmitted drug resistance to NRTIs, nNRTIs, and PIs

Among the 1307 HIV-1 specimens from INSTI-naive (948 ART-naive and 359 ART-experienced) and 63 raltegravir-experienced patients, the PR and RT genes were successfully amplified and sequenced in 1292 specimens (94.3%). The baseline demographic and clinical characteristics of the patients with PR and RT genes successfully sequenced were similar to those without analyzable RT/PR sequences (data not shown). Among the 6 samples from ARV-naive patients with INSTI major mutations, only one harboured M184V mutation (Supplementary Table 1), known to confer high-level resistance to lamivudine or emtricitabine; no other mutations related to non-nucleoside reverse-transcriptase inhibitors (nNRTIs) and protease inhibitors (PIs) were identified, which was significantly different from the prevalence of resistance mutations to nNRTIs (11.8%) and PIs (2.5%) observed in individuals without major mutations to INSTIs (Table 2). For the raltegravir-experienced patients, the prevalence of resistance mutations of HIV-1 sequences to any class of ART (96.6% vs 51.6%, *P* < 0.001), nucleos(t)ide reverse-transcriptase inhibitor (NRTIs) (96.6% vs 38.7%, *P* < 0.001), and multi-drug resistance (MDR) (69.0% vs 29.0%, *P* = 0.002) among patients with HIV-1 sequences harbouring INSTI mutations were all significantly higher than those without INSTI major mutations (Table 2).

When the current regimens used by the ART-experienced patients were analyzed, we found that zidovudine/lamivudine, abacavir/lamivudine, and tenofovir disoproxil fumarate (TDF)/emtricitabine or TDF and lamivudine were the major NRTI backbone antiretrovirals that were used in these patients (Table 2). The third agent used in these INSTI-naive patients usually included nNRTI (146/317, 46.1%) or PI (166/317, 52.4%), while in raltegravir-experienced patients, INSTI became the predominant third agent (42/61, 68.9%), followed by PI (15/61, 24.6%) and nNRTI (5/61, 8.2%). Nevertheless, in raltegravir-experienced patients infected with HIV-1 harbouring INSTI-related mutations, a significantly higher percentage of them received zidovudine/lamivudine as the backbone (67.9% v.s. 37.5%, *P* = 0.02) and INSTI as the third agent (85.7% v.s. 54.5%, *P* = 0.01). Accordingly, a significantly lower percentage of them used abacavir/lamivudine as the backbone (0% v.s. 31.3%, *P* = 0.002) and PI as the third agent (10.7% v.s. 36.4%, *P* = 0.01).

### Phylogenetic analysis

**Figure 3:**
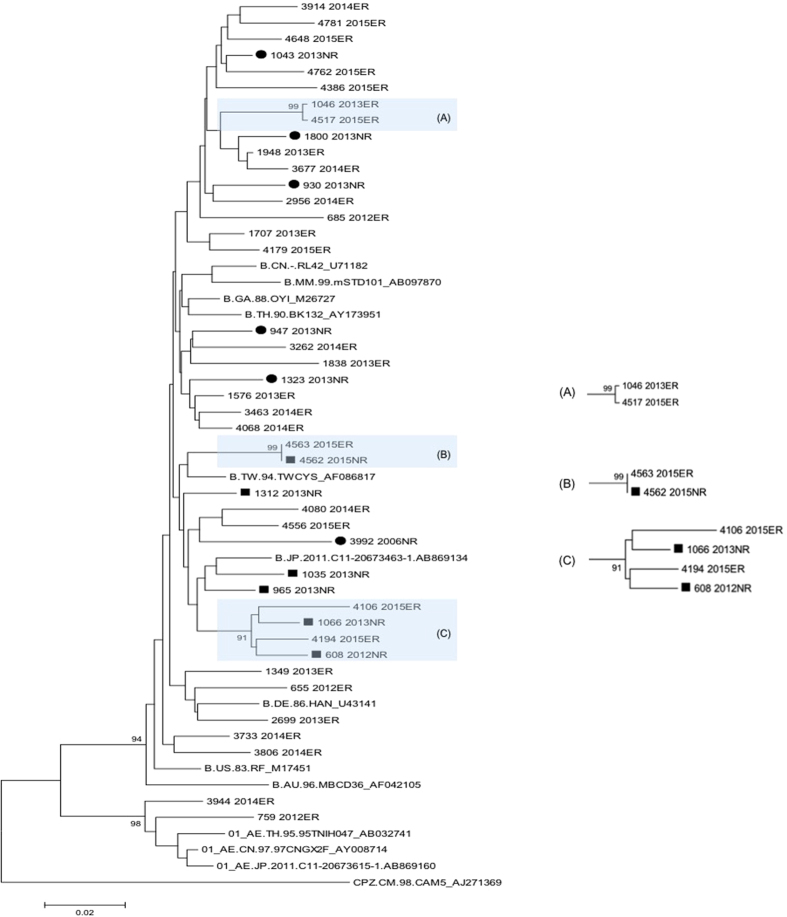
Phylogenetic analysis of integrase sequences amplified from INSTI-naive and INSTI-experienced patients. The 42 integrase sequences (6 from ART-naive, 6 from ART-experienced/INSTI-naive, and 30 from raltegravir-experienced patients) with major INSTI-related mutations were aligned with reference integrase sequences from database. ARV-naive was labeled as grey circle and the ARV-experienced/INSTI-naive was labeled as black square. The horizontal branch was drawn in accordance with their relative genetic distances. Bootstrap values greater than 700 of 1,000 replicates were considered significant and indicated at the nodes of the corresponding branches. The brackets at the right indicate the major sequence genotypes.

Phylogenetic analysis was conducted to determine whether there were clusterings observed among these integrase sequences amplified from INSTI-resistant sequences (Fig. 3). Three transmission clusters of subtype B were identified. One consisted of four sequences (cluster C), two from raltegravir-experienced patients and two from ARV-experienced/INSTI-naive patients. The other two transmission clusters were identified among the HIV-1 strains from raltegravir-experienced patients (cluster A), and raltegravir-experienced and ART-experienced/INSTI-naive patients (cluster B).

## Discussion

In this study conducted in Taiwan, where raltegravir was only available for clinical use since 2009, we demonstrate that the prevalence of major mutations to INSTI remains relatively low in INSTI-naive patients, 0.6% (6/948) among ART-naive and 1.7% (6/359) among ART-experienced/INSTI-naive patients, which is somewhat different from the absence of major mutations in ART-naive patients reported in the US and Europe^10^,^11^,^12^. Other than these major mutations, the prevalence of mutations at resistance-associated positions with a Stanford HIVdb score ≧ 10 was 5.3% and 38.1%, respectively, in ARV-naive and raltegravir-experienced patients.

In patients experiencing virological failure to the first generation of INSTIs, raltegravir and elvitegravir, three genotypic mutation pathways have been defined: Q148H/R/K (±G140S or E138A/K), N155H (±E92Q), and Y143C/H/R (±T97A). Except E92Q, the amino acid substitutions in the parenthesis representing the compensatory mutations that can help restore fully or partly viral fitness^14^,^15^,^16^. In general, the Q148 and N155 pathways are more frequently observed than the Y143 pathway in the raltegravir-experienced patients based on a French study (15.4% v.s. 19.1% v.s. 6.7%, respectively; N = 502)^17^. In a US study prior to the clinical use of dolutegravir, the prevalence of INSTI-mutations in 3012 specimens submitted for determinations of INSTI genotypic resistance, the Q148, N155, and Y143 pathway accounted for 6.5%, 6.5%, and 2.8%, respectively^12^. Of 30 subjects who failed INSTI-containing regimens in this study, 17 had Q148H/K/R mutations, 8 had N155H mutations, and 6 had Y143C/H/R mutations. In the 12 sequences with the Q148H mutation, all had G140S mutation, the predominant combination of INSTI-related resistance mutations as observed in raltegravir-experienced patients by Fourati *et al*.^17^. Among the 6 strains with Y143R mutations, 4 had T97A, which has been shown to increase Y143R/C-mediated resistance to raltegravir^18^.

The susceptibility of these INSTI-resistant sequences to the second generation of INSTI, dolutegravir, was also evaluated based on the results from *in vitro* studies^19^,^20^ and the clinical observation in the VIKING-3 clinical trial^21^. Significantly different from the study by Doyle *et al*. in the UK^11^, 1.59% and 19.05% of the sequences investigated in our study were defined as having high- or medium-level of resistance to dolutegravir, respectively. One potential reason for the difference is that our patients remained on failing regimens containing raltegravir for a longer period of time, which might lead to the accumulation of secondary mutations over time. In our study, a significantly higher percentage of these patients received zidovudine/lamivudine as the backbone (67.9% v.s. 37.5%, *P* = 0.02). Whether decreased tolerance to zidovudine/lamivudine may contribute to the risk of accumulating secondary mutations to raltegravir remains to be investigated. Besides the Q148 mutation combined with one or more of G140A/C/S, L74I and E138A/K/T was identified to reduce viral susceptibility to dolutegravir, two amino acid mutations, G118R and R263K, have been reported to confer low-level resistance to dolutegravir^22^,^23^. In our study, we did not identify any G118R substitution in our specimens, yet the prevalence of R263K was 0.31% and 1.64% in INSTI-naive and raltegravir-experienced patients, respectively.

Although raltegravir and elvitegravir have a relatively lower genetic barrier than PIs and most NRTIs, the prevalence of primary INSTI resistance mutations is relatively rare based on a few published reports^10^,^11^,^12^. No major INSTI-related mutations were observed in INSTI-naive patients by the CORONET study group and the ICONA Foundation study group, or in an epidemiological surveillance study using specimens collected by the European SPREAD programme in 2006–2007, before INSTIs were introduced into clinical care in Europe^10^,^11^,^13^. However, some minor mutations were observed. In the study by the European SPREAD programme, the prevalence of the integrase substitutions with a Stanford HIVdb score ≧ 10 to at least one INSTI was 4% of 278 specimens, which is comparable to 5.2% of 1307 specimens observed in this study. Casadella *et al*. who analyzed 56 specimens from the 278 specimens by 454 sequencing to detect the presence of scarce INSTI-related mutations found that the prevalence of mutations with Stanford HIVdb score ≧ 10 increased to 14.3%^10^. The impact of these mutations on the susceptibility of circulating HIV-1 strains to INSTIs is still unclear. However, some of the mutations are significantly associated with raltegravir exposure, such as L74M, T97A, E138K, V151I and G163R^11^. In our study, we also found that the prevalence of T97A, G140CAS, E157Q, or V151I was all significantly higher in HIV-1 strains from raltegravir-experienced patients than those from INSTI-naïve patients (Supplementary Table 3).

In INSTI-naïve patients, we observed that 0.7% of the sequences (N = 9) harboured the N155S/T mutation, which was not observed in any of the raltegravir-experienced patients in our study. N155S was first identified by *in vitro* passage experiment in the presence of raltegravir, and it has never been reported in sequences from patients receiving raltegravir^24^. Whether the presence of N155S/T only in INSTI-naive patients implicates its better transmissibility than N155H mutant viruses requires future investigations. Different from previous studies, we did not find significant association of L74M and G163R with raltegravir exposure and the prevalence of P145R was significantly higher in the strains from INSTI-naïve patients than those from raltegravir-experienced patients. These observations suggest that there are some sequence variations between HIV-1 strains worldwide, even for subtype B alone, and their influences on the emergence of INSTI-related mutations after initiation of INSTI-containing regimen warrant close monitoring.

In this study, an increased prevalence of INSTI-related genetic mutations was noted in 2013. It could be related to transmission of resistant strains among patients with risky behaviors since increasing incidence of HDV, HCV, syphilis, and *Entamoeba histolytica* have been observed in our MSM population in Taiwan^25^,^26^,^27^,^28^,^29^. Indeed, we identified 3 transmission clusters of subtype B in the phylogenetic analysis of integrase sequences with major mutations (Fig. 3). One cluster, cluster C, consisted of 4 sequences, 2 from raltegravir-experienced patients and 2 from ART-experienced/ INSTI-naive patients. Therefore, although raltegravir had not been available for clinical use in treatment-naive patients until 2012, we still observed a 0.9% of INSTI-related major mutations in the strains from INSTI-naive patients. The detection of INSTI-resistant major mutations in one ARV-naive patient in 2006 could be possibly related to clinical trial (BENCHMARK) of raltegravir in 2006 when a few patients were enrolled. Our study provides the first evidence that Q148H/K/R mutation can be transmitted from patients who failed raltegravir-containing regimens to raltegravir-naive patients. Three of the 6 treatment-experienced, but INSTI-naive patients were in two clusters of the phylogenetic analysis (filled squares in Fig. 3), suggesting that they might acquire the INSTI-resistant viruses from INSTI-experienced patients. In addition, the two study subjects in the cluster B were confirmed to be sexual partners. One of them, patient 4562, was naive to raltegravir treatment and was likely to acquire Q148H/K/R mutation from his partner, patient 4563, who had failed to respond to raltegravir-containing regimen with HIV-1 harbouring Q148H/K/R mutation.

The findings of this study should be interpreted with the necessary caution. First, the study was based on patients included mainly in hospitals in northern Taiwan, where the majority of the patients were MSM. While MSM have become the leading risk behavior for HIV infections and TDR is more prevalent among MSM in Taiwan^30^,^31^, our study results may not be generalized to the IDU and heterosexual populations. Second, the number of raltegravir-experienced patients included in this study remains small as compared to those of other studies, and continued surveillance studies are warranted when increasing use of raltegravir is likely and newer INSTIs are to be introduced into clinical use in the near future.

In conclusion, the prevalence of primary INSTI resistance of the HIV-1 strains identified from ARV-naive patients in Taiwan remains relatively low, which is comparable to the previous reports in high-income countries. Nevertheless, given our previous observation that TDR to other antiretrovirals remains high among MSM in Taiwan, close and vigilant monitoring of INSTI resistance is important before its widespread use in Taiwan.

## Materials and Methods

### Study setting

In Taiwan, cART has been provided free of charge since its introduction in April 1997, but routine drug-resistance testing has not been available to clinicians. By the end of December 2015, there were a total of 31,036 people diagnosed as having HIV infection in Taiwan, and about 70% of those who survived were receiving cART. The prevalence of transmitted drug resistance (TDR) in Taiwan has increased from 1999 to 2006, with an overall prevalence of 9.4%^30^. Decline in antiretroviral resistance mutations to NRTI and PI had been reported, followed by the increased use of more potent NRTIs and ritonavir-boosted PI combinations^31^. Nevertheless, due to financial constraints on the provision of free-of-charge access to cART, the Centers of Disease Control in Taiwan implemented regulations on the prescription of cART to antiretroviral-naive HIV-1-infected patients who received their first cART on 1 June 2012. Four categories of cART were defined: the first category consisted of nevirapine or efavirenz plus zidovudine/lamivudine (coformulated); the second category, nevirapine or efavirenz plus abacavir/lamivudine (coformulated); or TDF/emtricitabine (coformualted) or TDF and lamivudine; the third category, zidovudine/lamivudine plus PIs or raltegravir; and the fourth category, TDF/emtricitabine, TDF and lamivudine, or abacavir/lamivudine plus PIs or raltegravir. The fourth category required approval before prescription. Raltegravir was first introduced into Taiwan in 2006 when a few patients were enrolled in a clinical trial (BENCHMARK); however, it was not available to clinical use for ART-experienced patients until 2009; and in 2012, it was available for ARV-naïve patients to be combined with 2 NRTIs.

### Study subjects

HIV-infected Taiwanese patients receive HIV care according to the national treatment guidelines at designated hospitals around Taiwan by the Centers of Disease Control in Taiwan. Residual plasma samples for routine determination of plasma viral load (PVL) were obtained from HIV-1-infected patients seeking HIV care at the NTUH. A standardized case record form was used to collect information on demographics, antiretroviral medications, baseline CD4 lymphocyte count, and PVL. PVL and CD4 count were quantified with the use of Cobas Amplicor HIV-1 Monitor^TM^ Test, version 1.5, (Roche Diagnostics Corporation, Indianapolis, USA) and FACSFlow (Becton Dickinson), respectively. The study was approved by the Research Ethics Committee of the National Taiwan University Hospital, Taipei, Taiwan (REC registration number, 200905045R) and the experiment procedures were carried out in accordance with the approved guidelines. The patients who were linked to HIV care at the hospital gave written informed consent for determination of HIV-1 resistance mutations.

### Determination of drug resistance mutations

For those with confirmed HIV infection and linked to clinical care at NTUH, the genotypic resistance assays were performed retrospectively as described previously^30^,^31^. Antiretroviral resistance mutations were identified using the HIVdb program of the Stanford University HIV Drug Resistance Database (http://hivdb.stanford.edu; date last accessed, 10 November 2015), in accordance with the drug resistance mutation list of the International AIDS Society-USA Consensus Guidelines^32^. Multi-drug resistance (MDR) was defined as having genotypic resistance to more than one class of antiretroviral agents including INSTIs. Resistance-associated mutations (RAMs) were defined by the presence of at least one mutation included in the 2009 WHO surveillance drug resistance mutation list^33^.

To determine the resistance mutations related to INSTIs, an 884-bp fragment of *integrase* coding regions was PCR-amplified as described previously^34^. The PCR primer pair used was *pol*2950A (5′-TCA KCA CCT GCC ATC TGT TTT CC-3′)/2064A (5′-AYA ARG GRA TTG GAG GAA ATG AAC A-3′). The amplification condition was 35 cycles of 94 °C for 15 s, 55 °C for 1 min, 72 °C for 2 min, and a final extension at 72 °C for 7 min. The major INSTI mutations, which have been determined having a marked reduction of viral susceptibility to raltegravir and elvitegravir, include Y143C/H/R, Q148H/K/R, and N155H. Besides these three major mutations, the integrase substitutions with a Stanford HIVdb score ≧ 10 to at least one INSTI were included, such as H51Y, T66A/I/K, L74M, E92G/Q/V, Q95K, T97A, F121Y, E138A/K, G140S/C/A, Y143G/K/S/A, P145S, Q146P, S147G, V151A/L, S153F/Y, N155S/T, E157Q, G163 K/R, S230R, and R263K. Some amino acid substitutions (H51L/R, L74V, P145R, V151I, and S230N) that have been described in more than 2% of the study population in previous studies^11^,^13^,^35^ or this study were also included for analysis.

### Phylogenetic tree analysis

Reference sequences of various subtypes and recombinants were retrieved from the Los Alamos database (http://hiv-web.lanl.gov/seq-db.html). Sequences were aligned with the Clustal W listed in the MEGA (molecular evolutionary genetics analysis) analytical package (version 3.0)^36^ with minor manual adjustments. The phylogenetic trees were constructed by the neighbor-joining method based on the Kimura 2-parameter distance matrix listed in the MEGA software. Bootstrap values greater than 750 of 1,000 replicates were considered significant.

### Statistical Analysis

Data were analyzed using SAS 9.2 (SAS Institute, NC, USA). Categorical data were analyzed using chi-square or Fisher’s exact tests, as appropriate, and continuous variables were compared using the Wilcoxon test. All tests were two-tailed and a P value < 0.05 was considered significant.

## Additional Information

**How to cite this article**: Chang, S.-Y. *et al*. Prevalence of Integrase Strand Transfer Inhibitors (INSTI) Resistance Mutations in Taiwan. *Sci. Rep*. **6**, 35779; doi: 10.1038/srep35779 (2016).

## Supplementary Material

Supplementary Information

## Figures and Tables

**Table 1:** Baseline characteristics of study subjects.

		INSTI-naive	INSTI-naive		INSTI-exposed	*P* value		
Characteristics		N = 1307	Group A. ART-naïve N = 948	Group B. ART-experienced N = 359	Group C. ART-experienced N = 63	A vs B	A vs C	B vs C
**Male, n (%)**		1211/1263 (95.8)	881/907 (97.1)	330/356 (92.7)	58/61 (95.1)	<0.001	0.55	0.73
**Age, mean (SD), years**		33.0 (9.7)	31.4 (8.7)	37.2 (10.9)	34.4 (11.5)	<0.001	0.05	0.06
**Risk behavior, n (%)**	**MSM**	1035/1230 (84.1)	794/911 (87.2)	241/319 (75.5)	39/48 (81.3)	<0.001	0.24	0.39
	**IDU**	104/1230 (8.5)	61/911 (6.7)	43/319 (13.5)	2/48 (4.2)	<0.001	0.75	0.07
	**Heterosexual**	84/1230 (6.8)	53/911 (5.8)	31/319 (9.7)	7/48 (14.6)	0.02	0.05	0.43
	**Others**	7/1230(0.6)	3/911 (0.3)	4/319 (1.3)	0 (0)	0.16	>0.999	0.97
**HIV subtypes, n (%)**	**B**	1120 (85.7)	825(87.0)	295(82.2)	56(88.9)	0.03	0.67	0.19
	**C**	117 (9.0)	76(8.0)	41(11.4)	2(3.2)	0.05	0.24	0.05
	**CRF01_AE**	64 (4.9)	44(4.6)	20(5.6)	5(7.9)	0.48	0.36	0.62
	**Others**	6 (0.5)	3(0.3)	3(0.8)	0(0)	0.42	>0.999	>0.999
**PVL, mean (SD), log**_**10**_ **copies/mL**		4.74 (0.73)	4.81 (0.69)	4.53 (0.80)	4.44 (0.87)	<0.001	0.001	0.42
**PVL >5 log**_**10**_ **copies/mL, n (%)**		427/1290 (33.1)	328/941 (34.9)	99/349 (28.4)	12/62 (19.4)	0.03	0.01	0.14
**CD4 count, mean (SD), cells/μL**		299 (198)	315 (192)	256 (208)	312 (227)	0.0001	0.91	0.05
**CD4 count** < **200 cells/μL, n (%)**		405/1277(31.7)	249/938 (26.5)	156/339 (46.0)	21/60 (35.0)	<0.001	0.15	0.11
**Genotypic INSTI resistance, n (%)**		12 (0.9)	6 (0.6)	6 (1.7)	30 (47.6)	0.42	<0.001	<0.001

Abbreviations: ART, antiretroviral therapy; IDU, injecting drug users; INSTI, integrase strand transfer inhibitors; MSM, men who have sex with men; PVL, plasma HIV RNA load; SD, standard deviation.

**Table 2:**
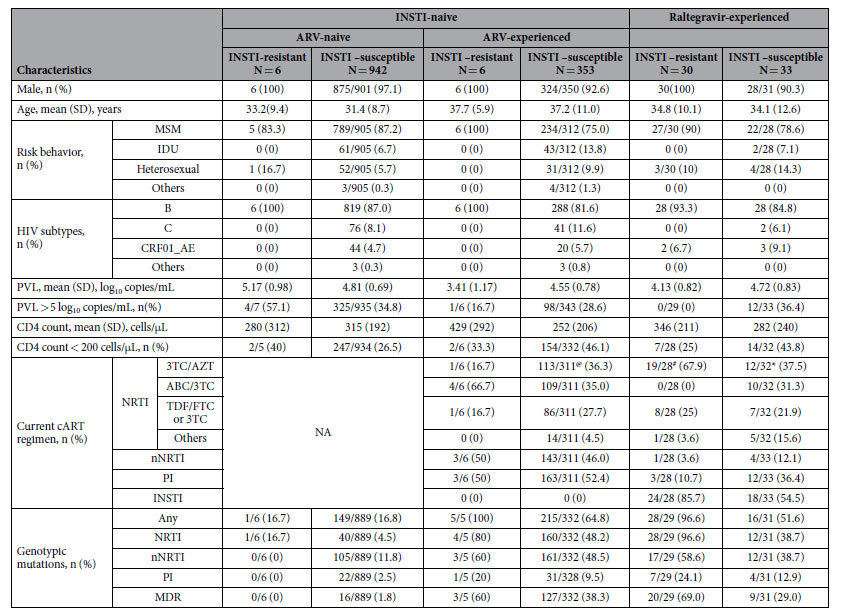
Characteristics of patients harbouring INSTI-related genotypic resistant mutations.

Abbreviations: 3TC, lamivudine; ABC, abacavir; ART, antiretroviral therapy; AZT, zidovudine; cART, combination antiretroviral therapy; FTC, emtricitabine; IDU, injecting drug users; INSTI, integrase strand transfer inhibitors; MDR, multi-drug resistance; MSM, men who have sex with men; PVL, plasma HIV RNA load; TDF, tenofovir disoproxil fumarate.

^@^Four patients used 3TC, AZT, and ABC; 3 patients used 3TC, ABC, didanosine (ddI), and boosted atazanavir; 1 patient used 3TC, ABC, ddI, and boosted lopinavir; 2 patients used 3TC, AZT, nevirapine, and ddI; 1 patient used 3TC, AZT, ddI, and boosted lopinavir.

^#^One patient used boosted darunavir, TDF, raltegravir, and etravirine; 1 patient used raltegravir and efavirenz.

^*^One patient did not use NRTI; 1 patient used 3TC, TDF, ABC, raltegravir; 1 patient used 3TC, ABC, TDF, and boosted darunavir.
