# Supplementary material for: Prevalence of Integrase Strand Transfer Inhibitors (INSTI) Resistance Mutations in Taiwan
Source: Sci Rep. 2016 Oct 25;6:35779. doi: 10.1038/srep35779 (PMC5078839; doi:10.1038/srep35779)
Supplement: Supplementary Information [file srep35779-s1.doc]

**Prevalence of Integrase Strand Transfer Inhibitors (INSTI) Resistance Mutations in Taiwan**

Sui-Yuan Chang^1,2^, Pi-Han Lin^1^, Chien-Lin Cheng^1^, Mao-Yuan Chen^3^, Hsin-Yun Sun^3^, Szu-Min Hsieh^3^, Wang-Huei Sheng^3^, Yi-Ching Su^1^, Li-Hsin Su^1^, Shu-Fang Chang^1^, Wen-Chun Liu^3^, Chien-Ching Hung^3,4,5^, Shan-Chwen Chang^3^

**^1^**Department of Clinical Laboratory Sciences and Medical Biotechnology, National Taiwan University College of Medicine, Taipei, Taiwan,

**^2^**Department of Laboratory Medicine, National Taiwan University Hospital and National Taiwan University College of Medicine, Taipei, Taiwan

**^3^**Department of Internal Medicine, National Taiwan University Hospital and National Taiwan University College of Medicine, Taipei, Taiwan

**^4^**Department of Medical Research, China Medical University Hospital, Taichung, Taiwan

**^5^**China Medical University, Taichung, Taiwan

Corresponding author: Chien-Ching Hung, Department of Internal Medicine, National Taiwan University Hospital, 7 Chung-Shan South Road, Taipei, Taiwan

**Supplementary Table 1. Summary of ARV-related genotypic mutations detected in INSTI-naive patients with major INSTI mutations**

| **Case Number** | **Date of specimen collected** | **ART-naive** | **INSTI** | | **NRTI**  **major mutations** | **nNRTI**  **major mutations** | **PI**  **major mutations** |
| --- | --- | --- | --- | --- | --- | --- | --- |
|  |  |  | **Major mutations** | **Minor mutations /polymorphism** |  |  |  |
| 3992 | 2006/11 | 1 | Q148QR |  |  |  |  |
| 930 | 2013/1 | 1 | Q148QR |  |  |  |  |
| 947 | 2013/1 | 1 | Q148QR |  |  |  |  |
| 1043 | 2013/3 | 1 | Q148QR |  |  |  |  |
| 1323 | 2013/5 | 1 | Q148QR | P145PR |  |  |  |
| 1800 | 2013/8 | 1 | Y143R | T97A, G163R | M184V |  |  |
| 608 | 2012/6 | 0 | Q148QR |  | K70R, M184V |  | M46I, I50L, V82A |
| 965 | 2013/1 | 0 | Q148QR |  | K65KR | Y181CY, F227CF |  |
| 1035 | 2013/3 | 0 | Q148QR |  | M184I | V108I, Y181C |  |
| 1066 | 2013/3 | 0 | Q148QR, N155NS | S230R |  | G190A |  |
| 1312 | 2013/5 | 0 | Q148QR | P145PR, L74V | T215S |  |  |
| 4562 | 2015/6 | 0 | Q148QR | H51HR |  |  |  |

Abbreviations: ART, antiretroviral therapy; INSTI, integrase strand transfer inhibitors

**Supplementary Table 2. Patterns of INSTI-related major/minor mutations in raltegravir-experienced patients**

|  |  | Q148 | | | N155H | Y143R |
| --- | --- | --- | --- | --- | --- | --- |
|  |  | H | K | R |  |  |
| Concomitant major mutations | N155H |  |  | 1 |  |  |
| Concomitant minor mutations | H51R |  |  | 1 | 1 |  |
|  | L74I |  |  |  | 3 |  |
|  | T97A |  |  |  |  | 3 |
|  | G140S | 10 |  | 2 |  |  |
|  | V151I |  |  |  | 1 |  |
|  | T97A+G140S | 1 |  |  |  |  |
|  | T97A+E157Q |  |  |  |  | 1 |
|  | E138K+G140A |  |  |  |  |  |
|  | V151I+E157Q |  |  |  | 1 |  |
| No minor mutations | |  |  | 2 | 1 | 2 |

**Supplementary Table 3. Distribution of INSTI-related major and minor mutations in study subjects**

|  | | **ARV-naive**  **N=948** | **ARV-experience/ INSTI-naïve, N=359** | **INSTI-experienced**  **N=63** | **Positive association with major mutations** |
| --- | --- | --- | --- | --- | --- |
| **Major mutations** | **Y143C/H/R*** | 1 (0.1) | 0 (0) | 6 (9.5) |  |
|  | **G148H/K/R*** | 5 (0.5) | 6 (1.7) | 17 (27.0) |  |
|  | **N155H*** | 0 (0) | 0 (0) | 8 (12.7) |  |
| **Minor mutations** | **L74M** | 20 (2.1) | 8(2.2) | 2 (3.2) |  |
|  | **E92V** | 1 (0.1) | 0 (0) | 0 (0) |  |
|  | **Q95K** | 4 (0.4) | 0 (0) | 0 (0) |  |
|  | **T97A*** | 2 (0.2) | 2 (0.6) | 6 (9.5) | Y143R/C/H |
|  | **E138AK** | 3 (0.3) | 0 (0) | 0 (0) | Q148H/R/K |
|  | **G140CAS*** | 0 (0) | 0 (0) | 13 (20.6) | Q148H/R/K |
|  | **Y143KGSA** | 0 (0) | 1 (0.3) | 0 (0) |  |
|  | **V151AL** | 0 (0) | 1 (0.3) | 0 (0) |  |
|  | **N155ST** | 6 (0.6) | 2 (0.6) | 0 (0) |  |
|  | **E157Q*** | 9 (0.9) | 2 (0.6) | 3 (4.8) | N155H |
|  | **G163K/R** | 5 (0.5) | 0 (0) | 0 (0) |  |
|  | **S230R** | 0 (0) | 1 (0.3) | 0 (0) |  |
|  | **R263K** | 2 (0.2) | 2 (0.6) | 1 (1.6) |  |
| **Polymorphisms** | **H51L/R@** | 11 (1.2) | 4 (1.2) | 2 (3.2) |  |
|  | **L74V** | 5 (0.5) | 4 (1.2) | 0 (0) |  |
|  | **P145R^#^** | 34 (3.6) | 2 (0.6) | 0 (0) |  |
|  | **V151I*** | 1 (0.1) | 2 (0.6) | 2 (3.2) |  |
|  | **S230N** | 206 (21.7) | 60(16.7) | 12 (19.0) |  |

Note: H51Y, T66AIK, F121Y, P145S, Q146P, S147G, S153FY were not detected in any of the study specimens.

* means a significant higher prevalence of INSTI-related mutations in RAL-experienced patients than those in RAL-naive patients.

# means a significant lower prevalence of INSTI-related mutations in RAL-experienced patients than those in RAL-naive patients.

@ means a significant higher prevalence of INSTI-related mutations in RAL-experienced patients than those in ART-naive patients.
